# Ventral stabilization of C1–C2 with additional fixation of C3 using screws and polymethylmethacrylate in a toy breed dog with atlantoaxial instability

**DOI:** 10.29374/2527-2179.bjvm008726

**Published:** 2026-09-03

**Authors:** Yuan Góes Ribeiro Campos, Murilo Henrique Dias da Silva, Isadora Villas Bôas Souza, Rafaela de Oliveira Cunha, Daniel de Pinho Alves, Carolina Camargo Zani Marinho, Adriano de Abreu Corteze, Paulo Vinícius Tertuliano Marinho

**Affiliations:** 1 Departamento de Medicina Veterinária, Instituto Federal de Educação, Ciência e Tecnologia do Sul de Minas Gerais, Campus Muzambinho, Muzambinho, MG, Brazil.; 2 Departamento de Medicina Veterinária, Universidade Federal de Lavras, Lavras, MG, Brazil.

**Keywords:** atlantoaxial subluxation, cervical stabilization, malformation, neurosurgery, subluxação atlantoaxial, estabilização cervical, malformação, neurocirurgia

## Abstract

Atlantoaxial instability (AAI) predominantly affects toy and miniature breed dogs, causing spinal cord compression and variable neurological deficits. This study aims to report a case of ventral stabilization of C1–C2 with additional fixation of C3 using screws and polymethylmethacrylate (PMMA) in a 5-month-old, 0.8 kg female Yorkshire Terrier with AAI. The patient presented with non-ambulatory tetraparesis after mild cervical trauma. Neurological examination revealed signs consistent with C1–C5 myelopathy and cervical pain. Radiographs and computed tomography of the cervical spine indicated dorsal displacement of C2 and severe spinal cord compression, confirming the diagnosis. After failure of conservative treatment, ventral stabilization was performed using 8 locked screws distributed between C1 and C3, combined with PMMA. Progressive clinical improvement was observed, with return to ambulation within 7 days and excellent neurological recovery at 165 days, with no complications during 12 months of follow-up. The stabilization technique using multiple screws and PMMA proved effective and safe in this case, enabling excellent neurological recovery even in the presence of severe neurological deficits. The inclusion of C3 in the stabilization process may have provided greater biomechanical resistance and reduced the risk of implant-related complications, representing a potentially relevant surgical approach in very small-sized patients.

## Resumo

A instabilidade atlantoaxial (IAA) acomete predominantemente cães de raças *toy* e miniatura, causando compressão medular e déficits neurológicos variáveis. Este estudo objetiva relatar um caso de estabilização ventral de C1–C2 com fixação adicional de C3 utilizando parafusos e polimetilmetacrilato (PMMA) em uma cadela Yorkshire Terrier de 5 meses de idade, pesando 0,8 kg, com IAA. A paciente apresentava tetraparesia não deambulatória após trauma cervical leve. O exame neurológico revelou sinais compatíveis com mielopatia C1–C5 e cervicalgia. Radiografias e tomografia computadorizada da coluna cervical evidenciaram deslocamento dorsal de C2 e compressão medular grave, confirmando o diagnóstico. Após falha do tratamento conservador, realizou-se estabilização ventral com 8 parafusos bloqueados distribuídos entre C1 e C3, associados ao PMMA. Observou-se melhora clínica progressiva, com retorno à deambulação em 7 dias e excelente recuperação neurológica aos 165 dias, sem complicações durante 12 meses de acompanhamento. A técnica de estabilização com parafusos múltiplos e PMMA mostrou-se eficaz e segura neste caso, possibilitando excelente recuperação neurológica mesmo diante de déficits neurológicos severos. A inclusão de C3 na estabilização pode ter conferido maior resistência biomecânica e reduzido o risco de complicações relacionadas aos implantes, representando uma abordagem cirúrgica potencialmente relevante em pacientes de porte muito pequeno.

## Introduction

Atlantoaxial instability (AAI) is a congenital condition that primarily affects small breed dogs, especially toy breeds, and is typically diagnosed in young animals under 2 years of age. It is often associated with congenital abnormalities of the odontoid process and ligamentous structures, although it may also occur secondary to cervical trauma (Slanina, 2016; Zani et al., 2015). Surgical stabilization is indicated in patients with severe neurological deficits or in cases refractory to conservative treatment (Platt & Da Costa, 2018). Although several techniques have been described in the literature, ventral stabilization has been associated with better clinical outcomes (Plessas & Volk, 2014), particularly techniques using multiple implants and polymethylmethacrylate (PMMA) (Aikawa et al., 2013; Sanders et al., 2004; Stout Steele et al., 2016).

The risk of implant-related complications, such as loosening and migration, remains a relevant concern, especially in young small breed dogs, which have immature bones and limited bone surface for fixation. In this context, strategies to improve biomechanical stability and the implant–bone interface have been proposed, including increasing the number and size of the implants (Barillaro et al., 2025; Platt et al., 2004). The inclusion of C3 in ventral stabilization may represent a technically advantageous modification to increase construct stability in dogs with AAI, with the potential to reduce the risk of implant failure, particularly in toy breed dogs (Cabreira et al., 2024). However, there is a scarcity of studies describing adjunctive fixation of C3 and its effects on surgical stability and clinical outcomes. Therefore, the present study aims to report the use of a ventral stabilization technique of C1–C2–C3 using multiple screws and PMMA in a young female toy breed dog with atlantoaxial instability.

## Case report

A 5-month-old female Yorkshire Terrier weighing 0.8 kg was referred to the Veterinary Hospital of IFSULDEMINAS – Muzambinho Campus with a 60-day history of progressive tetraparesis that began after low-intensity cervical trauma during play with another dog. General physical examination revealed no abnormalities. On neurological examination, non-ambulatory tetraparesis was observed, with greater impairment of the thoracic limbs, normal to increased spinal reflexes and extensor tone in all four limbs, as well as cervical hyperesthesia, findings consistent with C1–C5 myelopathy. Complete blood count and serum biochemistry were within reference ranges for the species, as was the electrocardiographic evaluation.

**Figure 1:**
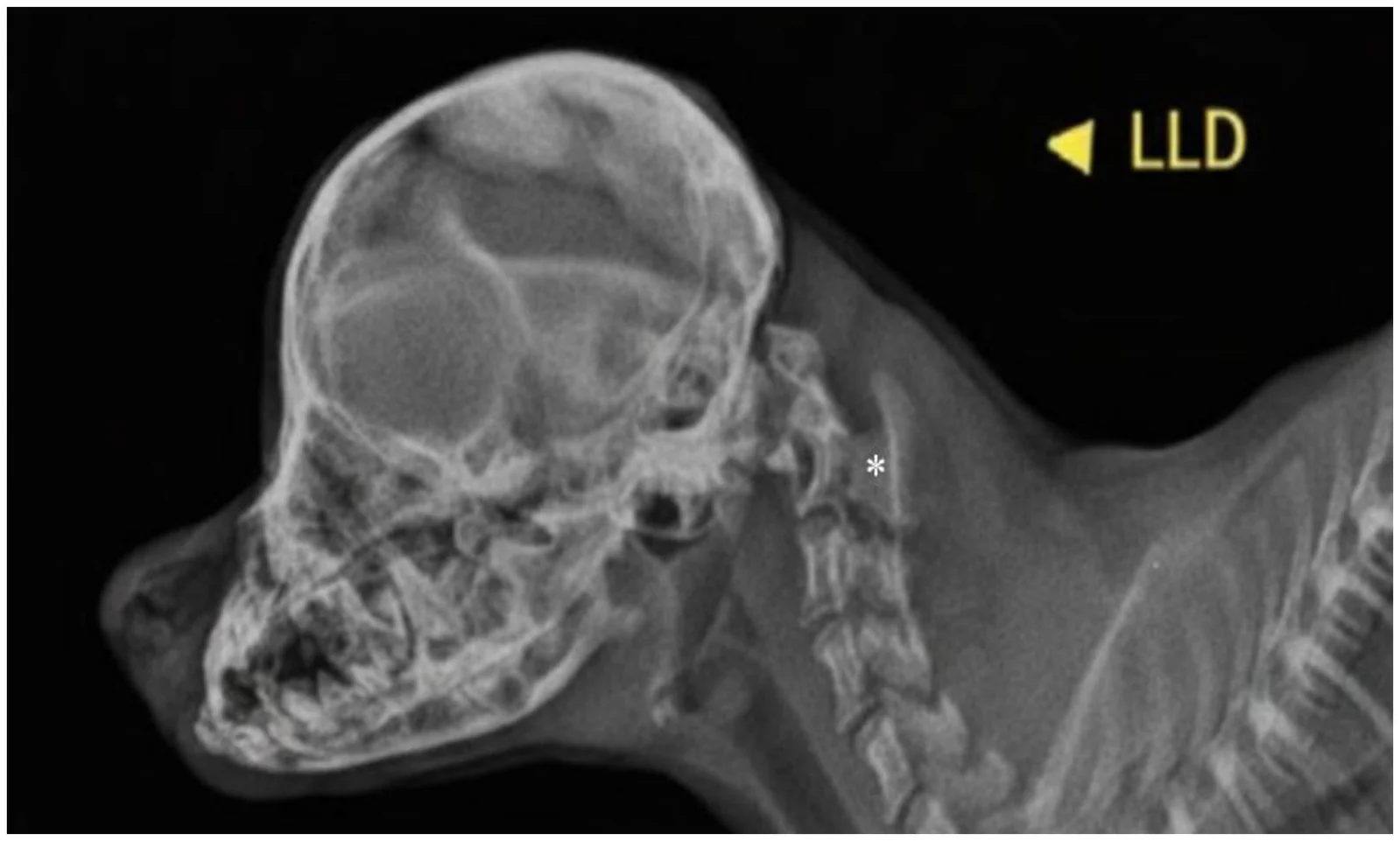
Right laterolateral radiograph of the cervical spine of a 5-month-old, 0.8 kg, intact female Yorkshire Terrier, treated at the Veterinary Hospital of IFSULDEMINAS – Muzambinho Campus, showing dorsal displacement of the axis (asterisk) relative to the atlas, consistent with atlantoaxial instability.

**Figure 2:**
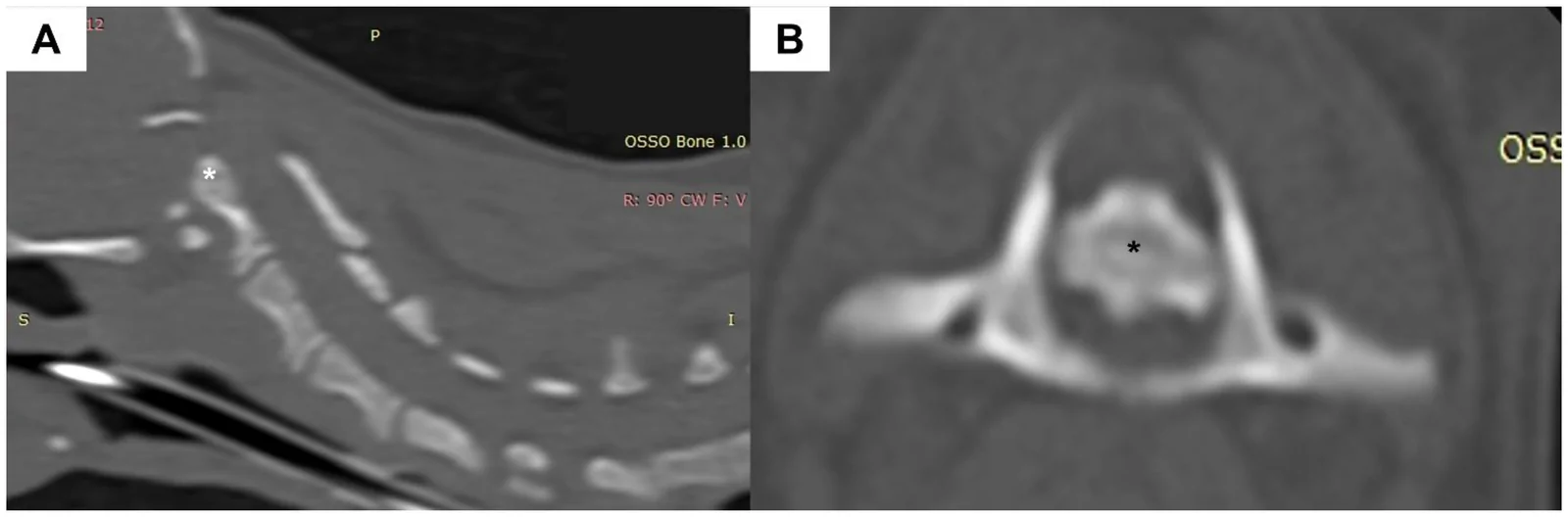
Computed tomography of the cervical spine of a 5-month-old, 0.8 kg, intact female Yorkshire Terrier, treated at the Veterinary Hospital of IFSULDEMINAS – Muzambinho Campus. A) Sagittal plane showing marked atlantoaxial subluxation with severe spinal cord compression by the odontoid process (white asterisk), associated with atlanto-occipital overlapping. B) Transverse plane showing projection of the odontoid process (black asterisk) and the cranial aspect of C2 into the vertebral foramen of C1, leading to reduced vertebral canal diameter.

Cervical spine radiography revealed dorsal displacement of C2 relative to C1, suggestive of AAI (Figure 1). Subsequently, computed tomography of the cervical region was performed for diagnostic confirmation and surgical planning. The examination revealed severe spinal cord compression caused by the displaced odontoid process, associated with hypoplasia of the odontoid process and the presence of an apical bone fragment with smooth, regular margins, consistent with a persistent centrum of the proatlas (Figure 2).

Initially, conservative treatment was instituted for 36 days with absolute rest, continuous use of a cervical splint, and medical therapy with pregabalin (5.0 mg/kg, PO, BID) and prednisolone (0.5 mg/kg, PO, BID), with gradual tapering over 21 days. Due to persistent cervical pain, amantadine (2.0 mg/kg, PO, SID) was added for 30 days. No significant clinical improvement was observed. Given the lack of response to conservative treatment, surgical ventral stabilization of C1–C2–C3 was performed using multiple screws and PMMA.

After the administration of pre-anesthetic medication, trichotomy of the ventral cervical region was carried out from the larynx to the manubrium. The patient was induced and positioned in dorsal recumbency with the neck extended and the thoracic limbs retracted caudally. Preoperative and definitive antisepsis was then performed using 2% chlorhexidine detergent, 10% povidone-iodine, and 0.5% alcoholic chlorhexidine.

**Figure 3:**
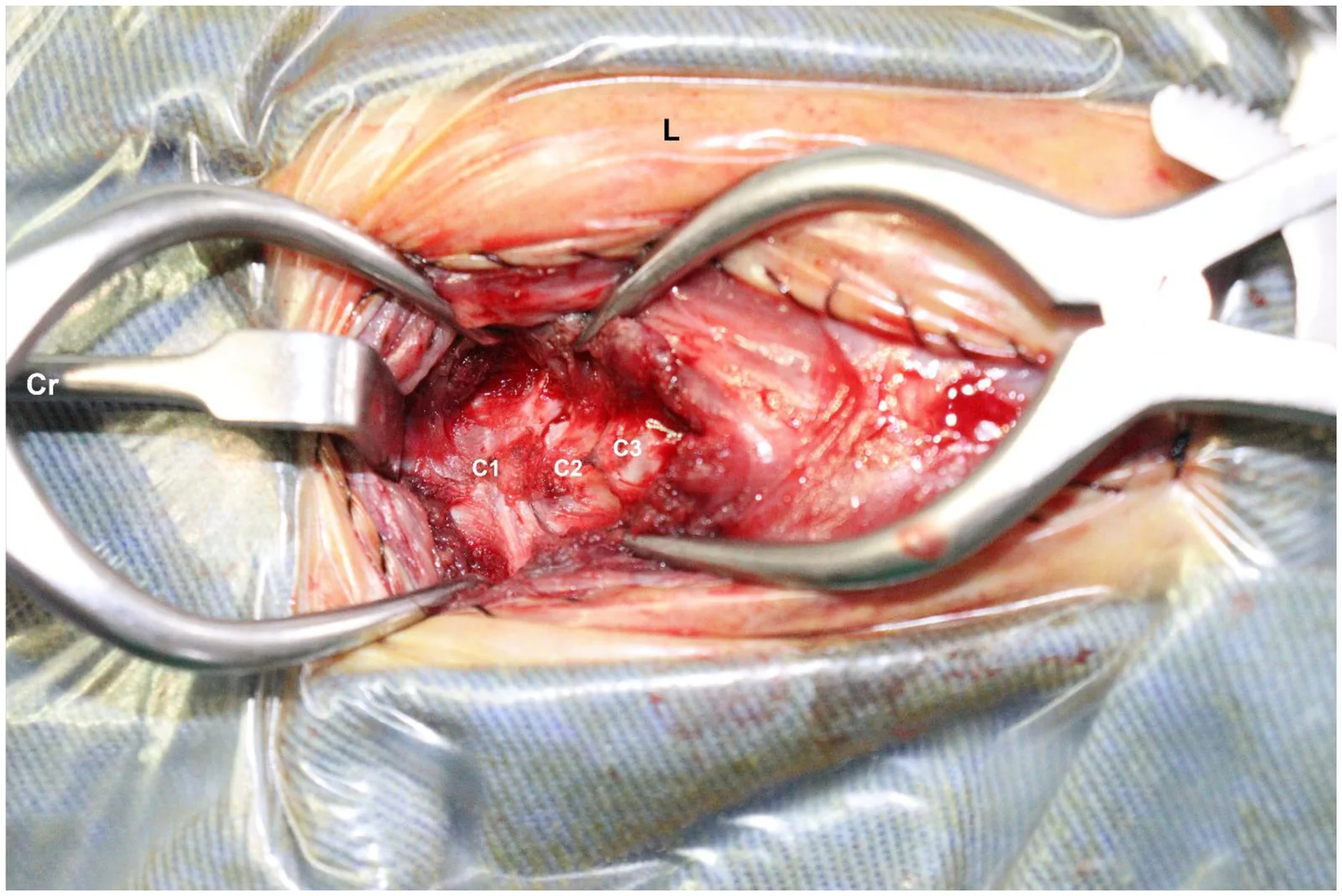
Intraoperative photograph of C1–C3 stabilization in a 5-month-old, 0.8 kg, intact female Yorkshire Terrier treated at the Veterinary Hospital of IFSULDEMINAS – Muzambinho Campus showing adequate exposure of the atlantoaxial joint and C3 vertebra after retraction of the *longus colli* muscle with Gelpi retractors. Cr: cranial; C1: atlas; C2: axis; C3: third cervical vertebra; L: left.

For the ventral approach to the atlantoaxial joint, access was achieved via a right parasagittal incision, as described by Shores and Tepper (2007). Dissection was performed between the sternothyroid and sternocephalic muscles, with identification of the right carotid sheath, which was retracted to the left together with adjacent structures, thus allowing exposure of the *longus colli* muscle. Thereafter, the tendinous insertions of the *longus colli* muscle on the ventral processes were incised and dissected, allowing it to be retracted and reflected using periosteal elevators. Exposure was maintained with Gelpi retractors placed cranially and caudally, allowing adequate visualization of the ventral surface from C1 to C3 (Figure 3). At 10-minute intervals, the retractors were relaxed to reduce the risk of iatrogenic injury due to compression of the recurrent laryngeal nerves.

**Figure 4:**
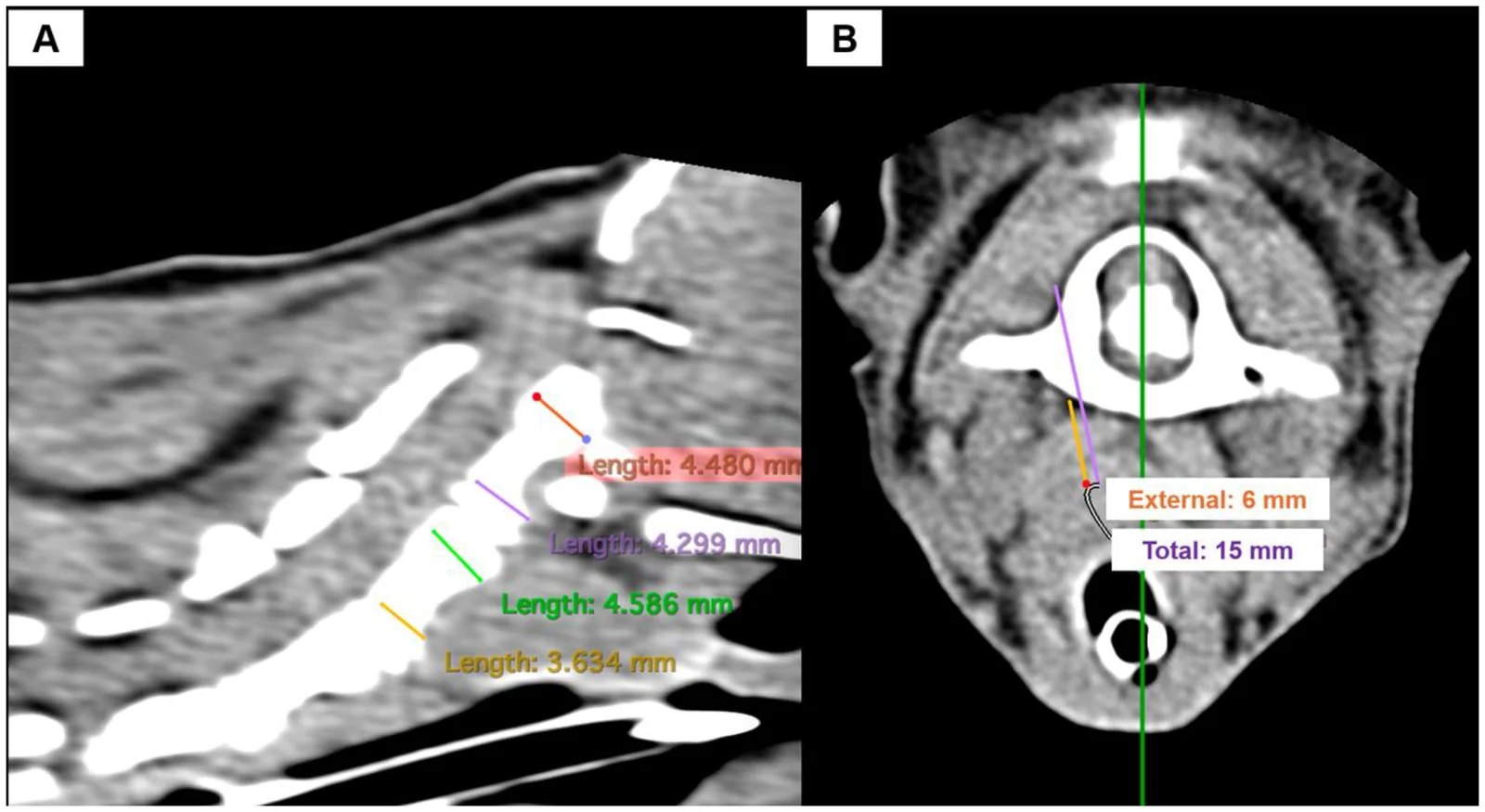
Computed tomography images used for preoperative planning in a 5-month-old, 0.8 kg, intact female Yorkshire Terrier treated at the Veterinary Hospital of IFSULDEMINAS – Muzambinho Campus, diagnosed with atlantoaxial instability. A) Measurement of the internal length of the C2 and C3 implants. B) Measurement of the bicortical screws in C1, evidencing an internal length of 9 mm and an external portion measuring 6 mm, totaling a 15 mm implant length.

Removal of the C1–C2 joint capsule and scarification with a No. 11 blade were conducted to facilitate joint fusion, followed by vertebral instrumentation from C1 to C3. Screw lengths were pre-determined based on measurements obtained from preoperative computed tomography images (Figure 4).

**Figure 5:**
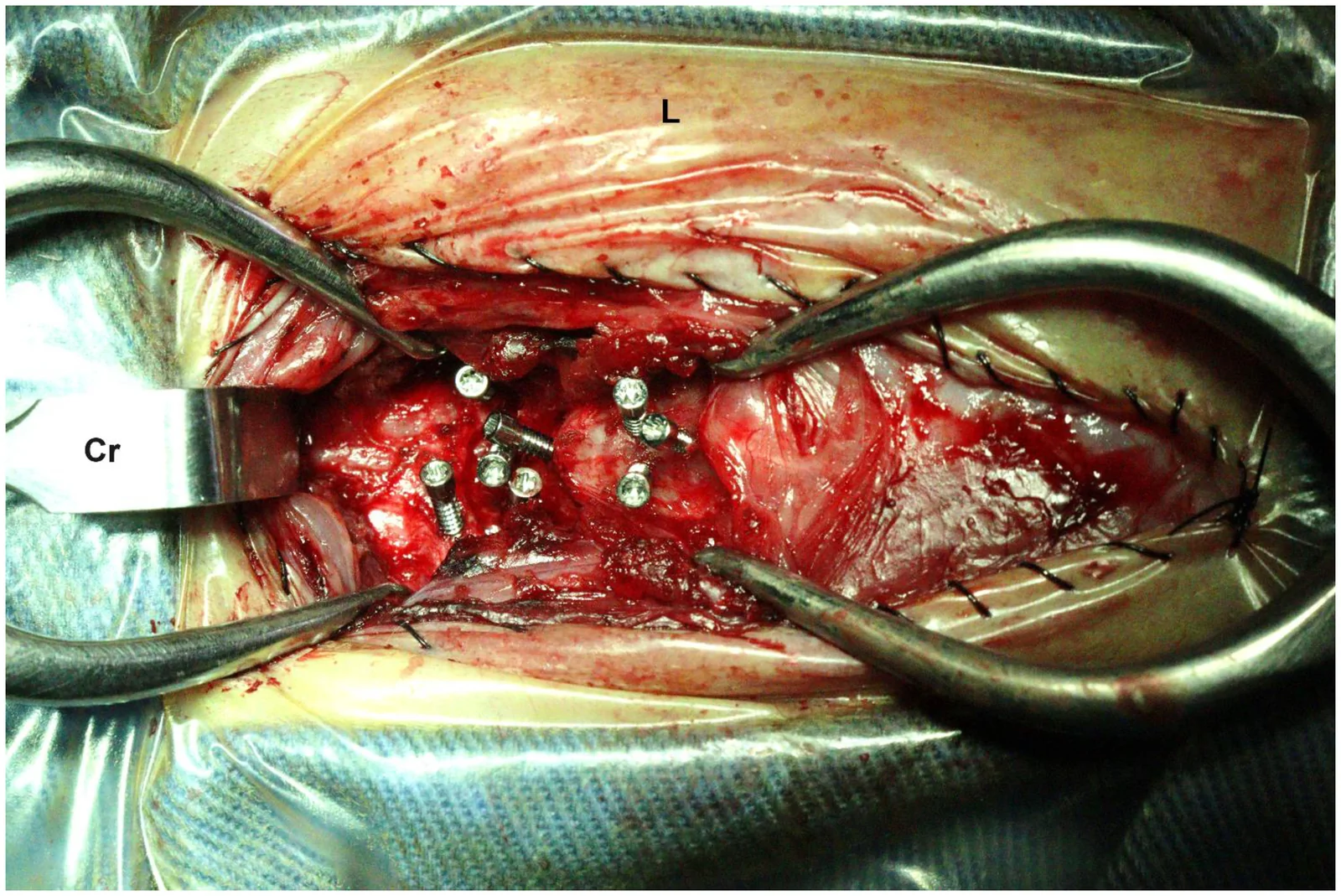
Intraoperative image of ventral C1–C3 stabilization with multiple screws and polymethylmethacrylate (PMMA) in a 5-month-old, 0.8 kg, intact female Yorkshire Terrier treated at the Veterinary Hospital of IFSULDEMINAS – Muzambinho Campus, evidencing the final aspect of the vertebral instrumentation. Cr: cranial; L: left.

Screw positioning and angulation were defined according to Sanders et al. (2004) and patient-specific 3D models of C1–C2. The same drilling protocol was adopted for all implants, with initial drilling using a 0.8 mm Kirschner wire, followed by drilling with a 1.3 mm drill bit. During all drilling procedures, a drill stopper was used to avoid inadvertent penetration into the vertebral canal. Eight 1.7 mm locking screws from the Fixin^®^ system were placed (Figure 5). Of these, two bicortical screws were inserted into the lateral masses of C1; three screws were placed in C2 (two bicortical into the cranial articular surfaces and one monocortical into the caudal metaphysis); and three monocortical screws were placed in C3.

**Figure 6:**
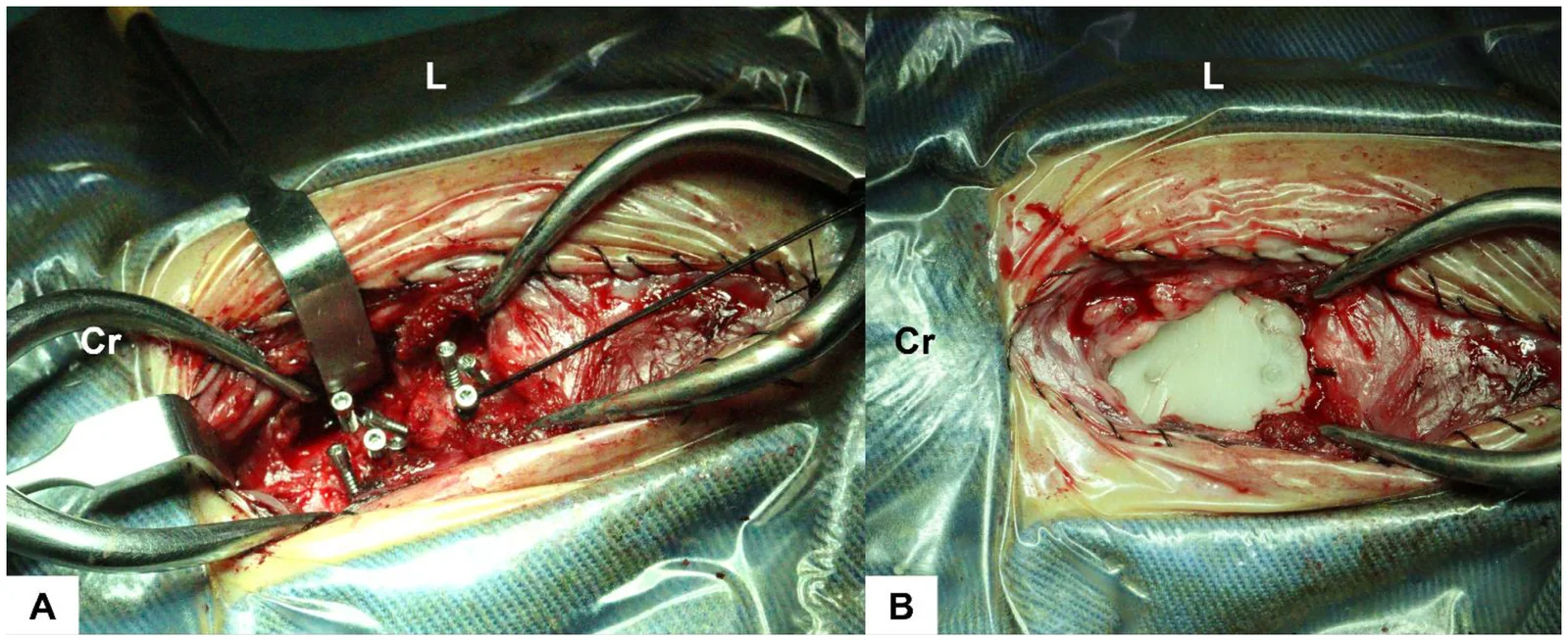
Intraoperative images of ventral C1–C3 stabilization with multiple screws and PMMA in a 5-month-old, 0.8 kg, intact female Yorkshire Terrier treated at the Veterinary Hospital of IFSULDEMINAS – Muzambinho Campus. A) Caudoventral traction of the nylon sutures attached to one of the cranial screws in C3 to reduce subluxation and realign the vertebrae. B) Final aspect of the surgical procedure after PMMA application and polymerization. Cr: cranial; L: left.

**Figure 7:**
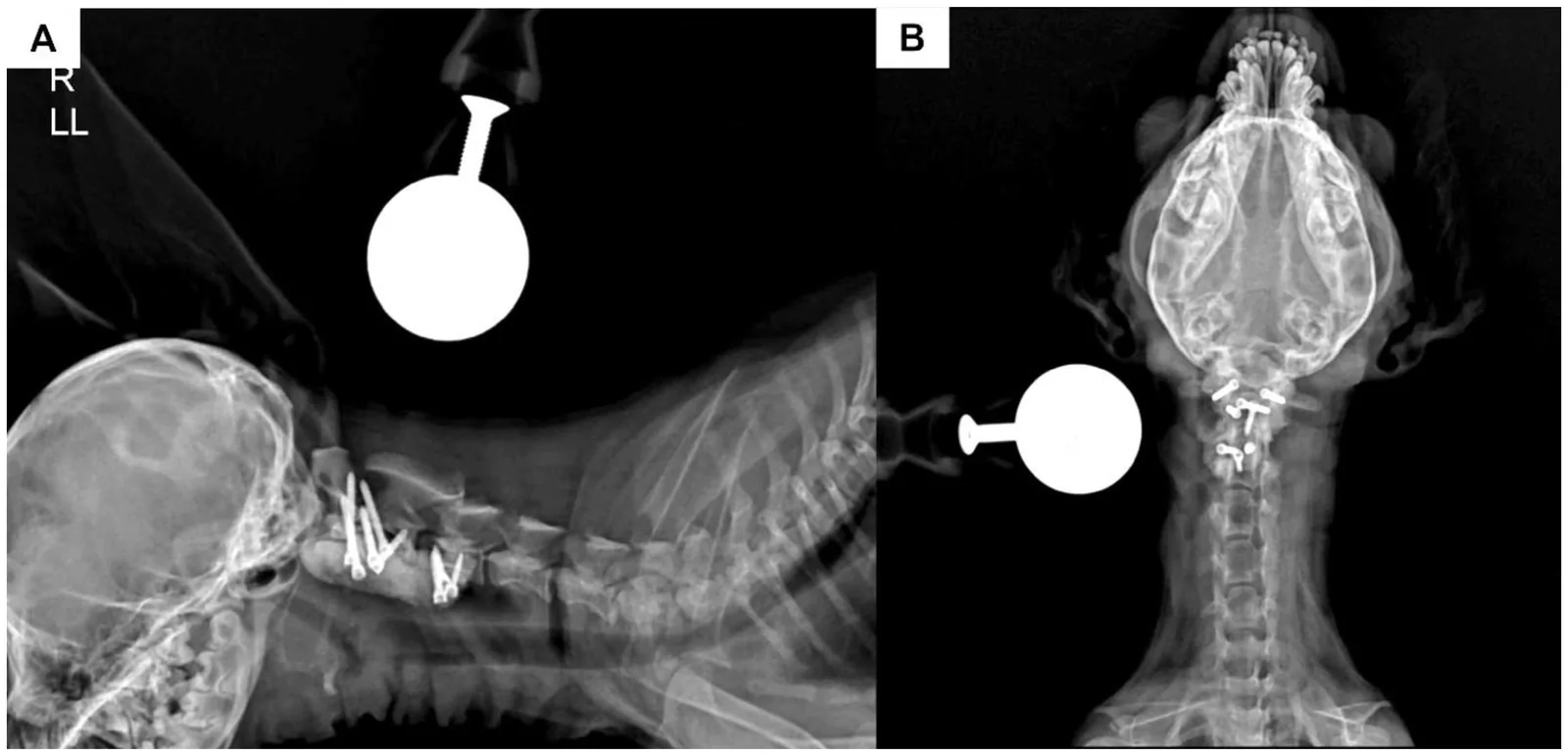
Immediate postoperative radiograph of the cervical spine of a 5-month-old, 0.8 kg, intact female Yorkshire Terrier treated at the Veterinary Hospital of IFSULDEMINAS – Muzambinho Campus, following ventral C1–C3 stabilization with multiple screws and PMMA, demonstrating adequate implant positioning, reduction of subluxation, and restoration of vertebral alignment. A) Right laterolateral projection. B) Ventrodorsal projection.

After implant insertion, two 2-0 nylon wires were fixed to the most cranial screw of C3 and tensioned caudoventrally (Figure 6A) to assist in vertebral realignment, a modification of the technique described by Platt et al. (2004). Subsequently, PMMA was applied, encasing the entire construct (Figure 6B). Following PMMA polymerization, the surgical site was routinely closed in layers. Immediate postoperative radiographs confirmed adequate implant positioning without invasion of the vertebral canal, as well as reduction of subluxation and restoration of vertebral alignment (Figure 7). After extubation, a cervical splint was applied for additional joint immobilization.

Postoperatively, the patient was hospitalized for 24 hours for monitoring and multimodal analgesic control, remaining stable with no changes in physiological parameters. The cervical splint was maintained for 6 weeks. Progressive clinical improvement was observed, with return to standing ability 5 days after the surgical procedure. At 7 days, the patient progressed to ambulatory tetraparesis, with significant improvement in motor function.

**Figure 8:**
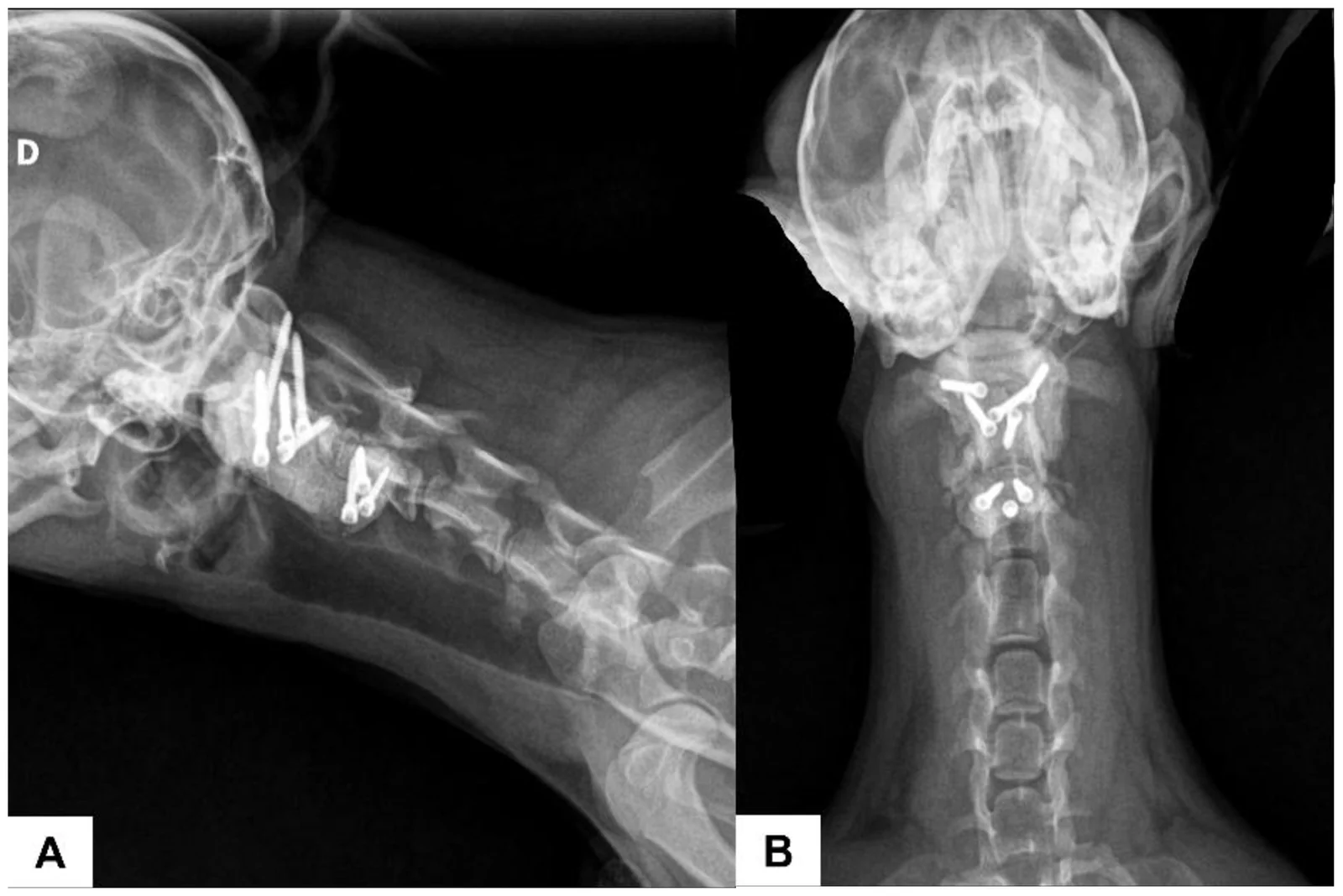
Postoperative 150-day follow-up radiograph of a 10-month-old, intact female Yorkshire Terrier treated at the Veterinary Hospital of IFSULDEMINAS – Muzambinho Campus, evidencing intact implants with no loosening or fracture, adequate vertebral alignment, and signs of bony ankylosis. A) Right laterolateral projection. B) Ventrodorsal projection.

Follow-up radiographs performed at 30, 60, and 150 days postoperatively demonstrated implant integrity, maintenance of vertebral alignment, absence of implant failure, and signs of vertebral fusion (Figure 8). At 165 days after the surgical procedure, the patient showed only mild residual proprioceptive ataxia in the pelvic limbs. On long-term follow-up, 12 months after the surgical procedure, the patient remained clinically stable with no neurological deficits or recurrence of clinical signs.

## Discussion

Minor trauma to congenitally unstable joints can trigger or worsen neurological signs due to dynamic spinal cord injury (Platt et al., 2004; Slanina, 2016). In the present case, the onset of clinical signs following low-intensity trauma possibly acted as a triggering factor for neurological signs in an animal with pre-existing subclinical instability, consistent with the findings reported by Beaver et al. (2000), who reported that 61% of traumatic AAI cases were associated with low-intensity traumatic events.

The asymmetric clinical presentation, characterized by non-ambulatory tetraparesis with greater involvement of the thoracic limbs, can be explained by the presence of an intramedullary lesion or by spinal cord compression exerted by the odontoid process at the midline, primarily affecting the upper motor neuron tracts located more internally and adjacent to the gray matter, destined for the thoracic limbs, known as central cord syndrome (De Lahunta et al., 2021; Ros et al., 2022).

The lack of response to conservative treatment is consistent with studies that describe clinical improvement rates ranging from 50–63%, with more favorable outcomes in patients with onset and duration of signs less than 30 days, as well as the presence of mild to moderate neurological deficits (Havig et al., 2005). In the present case, surgical stabilization was indicated due to the lack of response to conservative treatment, the severity of neurological deficits, and the degree of spinal cord compression.

Ventral stabilization with multiple screws and PMMA was the technique of choice due to the possibility of direct reduction of subluxation, adequate visualization of the articular surface, and precise implant placement (Sharp & Wheeler, 2005; Platt & Da Costa, 2018). Ventral stabilization techniques using screws and PMMA have demonstrated high clinical recovery rates, ranging from 84–94% (Barillaro et al., 2025; Platt et al., 2004; Sanders et al., 2004), which supports the selection of this approach in the present case to achieve greater biomechanical stability and lower risk of implant failure (Ferreira et al., 2023; Takahashi et al., 2017).

The favorable neurological outcome, with functional recovery over 12 months of follow-up, is consistent with the results described for ventral stabilization techniques using multiple screws and PMMA (Barillaro et al., 2025; Platt et al., 2004; Sanders et al., 2004). Prognostic factors such as young age and duration of signs present for less than 10 months may have contributed to the positive outcome, despite the initial severity of neurological deficits (Beaver et al., 2000).

Ventral stabilization in toy breed dogs remains challenging due to the limited bone stock available for fixation, skeletal immaturity, thinner cortical thickness, and greater bone fragility (Platt & Da Costa, 2018; Slanina, 2016). Implant failure rates can reach up to 44%, varying according to the duration of follow-up (Aikawa et al., 2013; Sanders et al., 2004). In this case, adjunctive stabilization of C3 was performed to increase construct stability and reduce the risk of implant failure, such as loosening and migration, especially considering the patient’s size and age. Although clinical studies specifically evaluating C1–C3 stabilization are scarce, Cabreira et al. (2024) described concomitant stabilization of C3 in a surgical revision case after implant failure, with maintenance of stability and satisfactory long-term neurological recovery.

Biomechanical studies have demonstrated that ventral constructs, particularly in the C1–C3 configuration, promote more uniform load distribution along the cervical spine, reducing cortical load at the atlantoaxial joint (Trębacz et al., 2026). Compared to stabilization restricted to C1–C2, extending fixation to C3 may increase the bone–implant interface, which favors better force distribution and decreases mechanical demand on the C1-C2 segment. Furthermore, it allows the placement of more screws, which may contribute to greater fixation security. Thus, stabilization involving C1–C3 may be a viable alternative in cases where there is a higher risk of implant failure or when the number of screws that can be safely inserted into C1–C2 is limited, such as in small patients with skeletal immaturity.

## Conclusion

Ventral stabilization of C1–C3 with multiple screws and PMMA proved to be an effective and safe technique for treating atlantoaxial instability in this case, resulting in excellent neurological recovery. Adjunctive fixation of C3 may represent a beneficial strategy to increase construct stability and reduce the risk of implant failure, especially in small patients with anatomical limitations that hinder conventional stabilization. However, additional biomechanical studies and long-term clinical studies with larger sample size are necessary to validate the clinical applicability and potential benefits of including C3 in atlantoaxial stabilization.
